# MR Imaging of Patients with Lateral Epicondylitis of the Elbow: Is the Common Extensor Tendon an Isolated Lesion?

**DOI:** 10.1371/journal.pone.0079498

**Published:** 2013-11-14

**Authors:** Liang Qi, Zheng-Feng Zhu, Feng Li, Ren-Fa Wang

**Affiliations:** 1 Department of Radiology, The First Affiliated Hospital of Nanjing Medical University, Nanjing, China; 2 Institute of Cardiology, Union Hospital, Tongji Medical College of Huazhong University of Science and Technology, Wuhan, China; 3 Department of Radiology, Tongji Hospital, Tongji Medical College of Huazhong University of Science and Technology, Wuhan, China; University Medical Center (UMC) Utrecht, The Netherlands

## Abstract

**Objective:**

To investigate whether an injury of the common extensor tendon (CET) is associated with other abnormalities in the elbow joint and find the potential relationships between these imaging features by using a high-resolution magnetic resonance imaging (MRI).

**Methods:**

Twenty-three patients were examined with 3.0 T MR. Two reviewers were recruited for MR images evaluation. Image features were recorded in terms of (1) the injury degree of CET; (2) associated injuries in the elbow joint. Spearman’s rank correlation analysis was performed to analyze the relationships between the injury degree of CET and associated abnormalities of the elbow joint, correlations were considered significant at *p<*0.05.

**Results:**

Total 24 elbows in 23 patients were included. Various degrees of injuries were found in total 24 CETs (10 mild, 7 moderate and 7 severe). Associated abnormalities were detected in accompaniments of the elbow joints including ligaments, tendons, saccussynovialis and muscles. A significantly positive correlation (*r* = 0.877,*p<*0.01) was found in injuries of CET and lateral ulnar collateral ligament (LUCL).

**Conclusion:**

Injury of the CET is not an isolated lesion for lateral picondylitis, which is mostly accompanied with other abnormalities, of which the LUCL injury is the most commonly seen in lateral epicondylitis, and there is a positive correlation between the injury degree in CET and LUCL.

## Introduction

Lateral epicondylitis, commonly known as “tennis elbow”, is the most common cause of discomfort or disability of elbow [1]. Lateral epicondylitis, caused by repeated contraction of the forearm extensor muscles, progressively results in micro-tearing with subsequent degeneration, immature repair, and tendinosis, particularly at initial part of the common extensor tendon (CET) [2], [3].

The diagnostic gold standard of lateral epicondylitis is essentially clinical examination. Radiographic film image and ultrasound are also helpful to clinical diagnosis, and MR imaging is not needed initially. However, when some symptoms are resistant to medical management [4], it is necessary to need an MRI scan, which can provide additional information about other abnormalities. Besides having excellent contrast resolution of soft tissue, high-resolution MR has ability to find subtle changes in the body, Accordingly, with this technique, our purpose is to investigate whether the injury of CET is associated with other abnormalities and find the potential relationships between these imaging features.

## Materials and Methods

The investigation conforms to the principles outlined in the Declaration of Helsinki. The trial was approved by the ethics committee of Tongji Medical College of Huazhong University of Science and Technology. Patients and controls provided written informed consent.

During 13-month period, 23 clinically diagnosed lateral epicondylitis patients (11 men, 12 women; range, 24∼59 years; mean age, 44 years) including 24 elbows underwent MRI examination. The duration of symptoms ranged from 1 week to 15 years. None of the patients had corticosteroid injection into the area of lateral epicondylitis within the preceding 3 months of MRI examination. Plain radiography had been performed to exclude the possibility of bony lesion. All patients underwent ultrasound assessment of the involved elbows.

A 3-Tesla MR system (SignaHDxt, GE Medical Systems, Milwaukee, Wisconsin, USA) with a dedicated surface coil was employed. Patients were performed in supine position, arms extended with the palms up and the elbows were placed in the center of the MR scanner as close as possible to achieve high image quality. Parameters of MR sequences are provided in Table 1.

**Table 1:** Parameters of MRI sequences.

Plane	Sequence	TR (ms)	TE (ms)	ETL	Matrix	BW (Hz)	FOV mm	Thickness (mm)	Gap (mm)
Coronal	T1 FSE	600	23	3	320×256	15	180	2	0.2
Coronal	T2 FS FRFSE	2380	48	12	320×256	31	180	2	0.2
Axial	T1 FSE	600	15	3	320×256	15	160	2	0.2
Axial	T2 FS FRFSE	2000	42	10	320×256	31	160	2	0.2
Sagittal	T1 FSE	600	23	3	320×256	15	180	2	0.2
Sagittal	T2 FS FRFSE	2380	48	12	320×256	31	180	2	0.2

Note:FSE = fast spin echo, FRFSE = fast recovery fast spin echo, BW = bandwidth, ETL = echo train length, FOV = field of view, FS = fat saturated, TR = repetition time, TE = echo time.

Acquisition date and participants identification were removed from all MR images. The data were assessed by two MSK radiologists with seventeen and twenty years of experience in interpreting cross-sectional images. The reviewers were blinded to all clinical information and were asked to record the following imaging features: (1) The injury degrees of CETs and ligaments: categorized as mild, moderate and severe (Table 2); (2) Injury of the muscles and bones: negative and positive (Table 3); (3) Effusion of joints: categorized as negative and positive (Table 3) [5]–[7]. Any discrepancies were settled by consensus.

**Table 2:** The classification of the CET and ligament injury.

Injury degree	CET	Ligament
0	Complete homogenous low intensity without thickness	Complete homogenous low intensity without thickness
Mild	Thickened tendon with internal focal increasing signal on fat-suppressed T2 image	Thickened ligament characterized by normal to hyperintensity and without interruption on fat-suppressed T2 image
Moderate	A fluid-filled gap affecting 20–80% of the thickness	Thinning of the ligament with hyperintensity within and surrounding the ligament
Severe	A fluid-filled gap affecting more than 80% of the thickness	A complete rupture and discontinuity of the fibers with fluid-like intensity

Note: CET = common extensor tendon.

**Table 3:** The classification of injury of the bone, muscle and joint effusion.

Injurydegree	Muscle	Bone	Joint effusion
I	Normal	Normal	Normal
II	High signal intensity	High signal intensity	The fluid increased

Spearman’s rank correlation analysis was performed to analyze relationships between the injury degree of the CETs and the associated abnormalities of elbow joints, correlations were considered significant at *p<*0.05. Statistical analysis was performed using SPSS (Statistical Packages for the Social Sciences) version 13.

## Result

**Figure 1:**
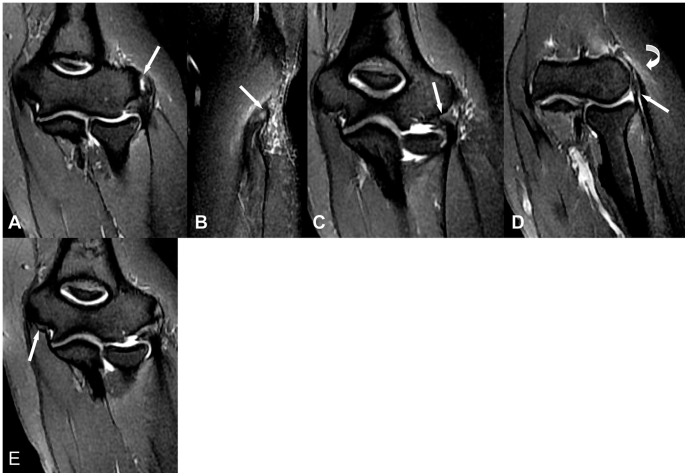
A 36-year-old female with left elbow pain about 1 month. (A,B) Coronal fat-suppressed FSE T2-weighted image shows abnormal fluid signal intensity affecting about 50% of the CET thickness (white arrow), demonstrating moderate injury. (C) Coronal fat-suppressed FSE T2-weighted image shows thickening and abnormal mildly increased signal intensity within the LUCL origin (white arrow), which suggests mild injury. (D) Coronal fat-suppressed FSE T2WI shows thickening and mildly increase signal intensity within the RCL (white arrow), demonstrating mild injury. Intramuscular edema shows as high-signal-intensity focus is seen within the extensor carpi muscle (white curve arrow ). (E) Coronal fat-suppressed FSE T2-weithted image shows mildly increased signal intensity within the proximal MCL (white arrow), a finding suggestive of mild injury.

**Figure 2:**
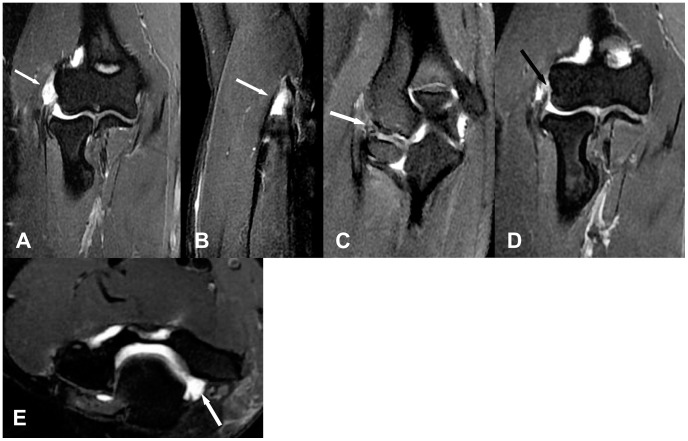
A 40-year-old female with right elbow pain about 3 months. (A,B) Coronal and Saggital fat–suppressed FSE T2-weighted image shows abnormal fluid signal intensity affecting more than 80% of the CET thickness, which suggests severe injury (white arrow). (C) Coronal fat-suppressed FSE T2-weighted image shows thinning and line-like high signal intensity across the LCUL origin, a finding suggestive of severe injury (white arrow). (D) Coronal fat-suppressed FSE T2-weithted image shows complete absence of the proximal fibers of the RCL (black arrow). demonstrating full-thickness tear of the RCL (white arrow). (E) Axial fat-suppressed FSE T2-weighted image shows joint effusion (white arrow).

Total 23 patients with lateral epicondylitis were included, 6 patients presented with lesions in left elbows, 16 in right elbows, and 1 in both elbows. From MRI, various degrees of injuries were found in total 24 CETs (10 mild, 7 moderate and 7 severe. Fig. 1A,B 2A,B). With regard to associated complications, the imaging patterns in these 23 patients were characteristic with multiple injuries, including radial collateral ligament (RCL) injury in 8 elbows (Fig. 1D,2D), medial collateral ligament (MCL) injury in 3 elbows (Fig. 1E), extensor muscle injury in 7 elbows (Fig. 1D), bone change in 6 elbows, joint effusion in 6 elbows (Fig. 2E) and anconeus muscle injury in 7 elbows (Table 4). In all these patients, high incidence of LUCL injury (91.67%, 22/24) was concomitantly found, 9 of them presented with mild, 6 with moderate and 7 with severe injuries (Fig. 1C and 2C). Spearman’s test showed a significantly positive correlation in injuries of the CET and LUCL (correlation coefficient *r* = 0.877,*p<*0.01. Table 5).

**Table 4:** Results of injuries in patients with lateral epicondylitis.

Injury degree	CET	LUCL	RCL	MCL	Extensor muscle	Anconeus muscle	Bone	Joint effusion
0	–	2	16	21	–	–	–	–
I	10	9	5	1	17	17	18	18
II	7	6	2	1	7	7	6	6
III	7	7	1	1	–	–	–	–

Note: CET = common extensor tendon, LUCL = lateral ulnar collateral ligament, RCL = radial collateral ligament, MCL = medial collateral ligament.

**Table 5:** Correlation of the CET injury with associated abnormalities of elbow.

	CET injury	
Associated abnormalities	*R*	*p*
LUCL	0.877	<0.01
RCL	0.314	0.136
MCL	0.349	0.096
Extensor muscle	0.106	0.622
Anconeus muscle	−0.113	0.599
Bone	0.319	0.129
Joint effusion	−0.015	0.945

Note: CET = common extensor tendon, LUCL = lateral ulnar collateral ligament, RCL = radial collateral ligament.

## Discussion

Lateral epicondylitis is generally characterized by a progressive degeneration, immature repair, and tendinosis at initial part of the CET [8], [9]. However, in our study, we demonstrat that injury of the CET is not an isolated lesion for lateral epicondylitis, which is mostly accompanied with other abnormalities. The results indicate that progressive injury of the CET is strongly associated with complicated injury in the LUCL.

The LUCL originate from the lateral epicondyle as a continuation of the RCL, running along the lateral and posterior aspects of the radius to insert on the tubercle of the supinator crest of the ulna, the function of the LUCL contributes to ligamentous constraint against varus stress, and disruption of the LUCL results in posterolateralrotatory instability of the elbow [3], [10]. In our study, lateral epicondylitis was most commonly associated with LUCL injury. 22 (92%) of 24 elbows showed abnormalities in the LUCLs on MR images. Spearman’s rank correlation analysis showed positive correlation between the injury of CET and LUCL, which means the more severe damage in the CET, the more severe injury in the LUCL. The previous papers reported that about 4–12% of patients with lateral epicondylitis undergo operative intervention [11]. The technique involves debridement the diseased tendon and cortication of the bone. If moderate or severe injury of the LUCL is not realized before the surgery, it may lead to further destabilization of the elbow [12]. Therefore, the patients with lateral epicondylitis, especially moderate and severe lateral epicondylitis, should be recommended for further MR examination to evaluate the extent of the LUCL injury.

Besides injury of LUCL, lateral epicondylitis could be associated with other abnormalities such as injuries of RCL, MCL, CET, bone, joint effusion and anconeus muscle. However, the results showed that there was no correlation between the injury of the CET and these other abnormalities. The RCL originates from the lateral epicondyle anteriorly and fascia of the supinator muscle. Thickness and tear of RCL has been identified in association with severe lateral epicondylitis [4]–[6]. In our study, 8 patients presented with injuries of RCLs, 6 of them were found in severe lateral epicondylitis. the RCL and LUCL share a common origin, thus, the RCL is also the stabilizers of the elbow. Injury of the RCL can lead to lateral instability of the elbow as well [13]. The MCL comprises three ligamentous bands: the anterior bundle, posterior bundle, and oblique band. The MCL is prone to concurrent injury with medial epicondylitis [14], [15], which is rare in lateral epicondylitis [16]. In our study, only 3 patients were accompanied with injury of the MCL, and all these lesions occurred in severe lateral epicondylitis. Individuals with injury of MCL present with medial instability of the elbow. The treatment of MCL injury is initially conservative and consists of activity modification. However, the current studies reported that most patients involved in high-level throwing activities do not respond well to conservative therapy, these individuals need further treatment with reconstruction of the MCL [16]. As a result of overuse of the wrist extensor muscle in patients with lateral epicondylitis, associated intramuscular edema may be seen in the common extensor muscle. Also this sign was demonstrated in 7 cases of our study, which showed feather-like high signal intensity within the common extensor muscle. The injury of muscle often occurred in acute lateral epicondylitis [17]. In our study, 3 of 7 patients with abnormalities of muscles had less than half a year’s clinical history, 3 patients had only 2 to 3 weeks. In Thornton’s study [18], lateral epicondylitis was accompanied with bone marrow edema in lateral epicondyle. In our study, the injury bones of elbows were involved in 6 patients, 4 of whom showed focal avascular necrosis and osteochondritisdissecans in the radial head, the rest were in the capitellum, and none of them showed abnormality in lateral epicondyle. Joint effusion can be seen in lateral epicondylitis. In our study, there were 6 (25%) of 24 elbows accompanied with joint effusion. The anconeus muscle is a small triangular muscle behind and below the elbow joint, It’s function is still controversial [19]. In Coel’s and Degreef’s studies [20], [21], which reported that chronic epicondylits was usually associated with the anconeus muscle injury. However, in our study, only 7 of 24 elbows had high signal intensity in the anconeus muscle. The reason may be that there were not only chronic but also acute epicondylitis patients in our study.

A limitation of our study is that pathologic specimens were not available for direct comparison with MRI findings since none of the patients underwent surgery, and consequently there is no pathologic gold standard of our cases; Another limitation was that the sample size was not large enough and the variables is a little bit more, in future work, we need to further expand the sample size.

In conclusion, injury of the CET is not an isolated abnormality of lateral epicondylitis, while, mostly accompanied with other pathological changes. Of all the accompany abnormalities, the LUCL injury is the most commonly seen in lateral epicondylitis, and there is a positive correlation between the injury degree of the CET and LUCL.
